# Using Evidence-Based Learning Strategies to Improve Medical Education

**DOI:** 10.1007/s40670-023-01798-9

**Published:** 2023-05-06

**Authors:** Christopher R. Madan

**Affiliations:** School of Psychology, University of Nottingham, Nottingham, UK

**Keywords:** Learning strategies, Retrieval practice, Spaced practice, Desirable difficulties

## Abstract

Medical education research has been adopting principles from psychology to improve student learning. Here is an overview and illustrative examples of six evidence-based learning strategies that have been thoroughly researched and validated in the psychology literature: spacing, interleaving, retrieval practice, elaboration, dual coding, and concrete examples. For each of these, their use within medical education and considerations that may influence efficacy are discussed. Medical education researchers should collaborate more with psychology researchers in transdisciplinary teams to better implement these strategies and more directly benefit from advances made in the psychology literature.

Medical education is associated with learning a massive amount of content over a few years. Without explicit instruction, students use very basic learning strategies such as re-reading and paraphrasing of notes [1], rather than much more effective evidence-based learning strategies that have been refined through psychology research over the last few decades. When taught these more evidence-based strategies, students found them more satisfying and had more confidence in their studying habits, as well as performed better [1–3]. Here are outlines of these learning strategies, the evidence that supports them, and indications of where further research is needed.

## Six Evidence-Based Learning Strategies

Though many learning strategies have been proposed and evaluated in the psychology literature, six have emerged as being particularly effective [4–6]. These strategies are (1) *spaced practice*, which involves distributing studying over time for better retention; (2) *interleaved practice*, which alternates between different topics to deepen understanding; (3) *retrieval practice*, a method that prioritizes active recall over passive review; (4) *elaboration*, the process of associating new information with existing knowledge; (5) *dual coding*, which utilizes both verbal and visual representations to support learning; and (6) using *concrete examples* to help students grasp abstract principles. These six strategies can each be grouped into pairs, based on their influence on the timing of study, how to study, and by being content-dependent strategies.

### Timing of Study

The first strategy is *spaced practice*, the principle of distributing studying over time. As an example, Jamie has an internal medicine exam in two months. With respect to other commitments, Jamie is willing to spend eight hours studying between now and the exam. Three possible approaches for allocating these hours would be as follows: (1) Four hours each over a Saturday and Sunday on the coming weekend, soon after the material was initially taught. (2) Four hours a day over the weekend just before the exam, right before it is needed. (3) Two hours per weekend over four of the weekends between now and the exam. Spaced practice studies suggest that the third option would yield the best results, particularly over a longer period [7, 8]. For instance, there may be a later cumulative exam or further content that would build on this material.

*Interleaved practice* is a related learning strategy. Continuing the same example with Jamie and the internal medicine exam, the content evaluated may include learning about twenty common medical presentations (e.g., abdominal pain, cough, headache). Studying each of these could be uniformly spaced within the designated eight study hours. But, if Jamie instead alternated between the twenty presentations, revisiting them in each study session in an alternating manner, learning outcomes would be improved [9, 10]. Moreover, through alternating between topics, content is often learned at a deeper level and a greater understanding of the relationship between concepts is achieved [11].

### How to Study

What should Jamie do during the designated study hours? Re-reading and note-taking are options, but a better option is *retrieval practice*, our third strategy. Here being tested is the priority, often using flashcards of definitions or practice multiple-choice questions. The more experience a student has with retrieving information from memory, the easier it will be to do so again during the summative test [12, 13]. Teachers can help facilitate this type of studying by including low-stakes quizzes in preparation of more substantial exams [14].

What should Jamie do during the designated study hours if further time allows? Learning more about the requisite topics will also enhance learning, otherwise referred to as *elaboration*. For Jamie, this could be done by engaging in wider reading through other textbooks and study materials that provide additional content beyond the level intended to be assessed. By associating the core content with further knowledge, the central details will remembered better [15]. Building off of one of the suggestions for retrieval practice, flashcards made by the student are more effective than pre-made flashcards—and would also be an ideal activity to encourage students like Jamie to engage in elaboration.

### Content-dependent Strategies

If Jamie encounters a topic that is relatively abstract, using both words and images to represent the idea can aid in retention. This strategy is referred to as *dual coding*, due to the involvement of multiple representations to support learning, such as verbal and pictorial, to support learning. This may be useful, for instance, when Jamie is learning about cellular physiology and how sodium–potassium pumps maintain the cell membrane potential; using diagrams along with textual descriptions may help improve understanding and retention.

Finally, *concrete examples* can benefit learning. When Jamie needs to learn abstract principles, a variety of specific examples can help demonstrate the underlying general principles. This article as a whole is intended to provide concrete examples. Within medical education training, simulations can serve this role well for Jamie [16]. As another example, when Jamie is learning about the complex concept of pharmacokinetics, concrete examples of how specific drugs interact within the body can help solidify understanding. By studying real-life scenarios, such as how acetaminophen is metabolized and eliminated, Jamie can better grasp the general principles of drug absorption, distribution, metabolism, and excretion.

### Challenges in Implementing Evidence-based Learning Strategies

A limitation of these six strategies is that they are often perceived by students as being more difficult and resulting in worse performance in immediate assessments, even though learning is far superior in the final assessments [9, 13, 17]. Due to this metacognitive discrepancy, these learning approaches have sometimes been referred to as *desirable difficulties*.

Moreover, there are other reasons why educators might struggle to incorporate these practices into their teaching, in addition to student resistance. One such reason could be the conventional and conservative nature of education, which often emphasizes traditional methods such as lectures, note-taking, and rote memorization. This can make it challenging for educators to deviate from the norm and adopt newer, evidence-based strategies. Additionally, teachers may face time constraints, lack of resources, or institutional resistance that make it difficult to implement these strategies effectively. To overcome these challenges, professional development programs and supportive educational policies can play a crucial role in equipping educators with the knowledge, skills, and resources necessary to adopt evidence-based learning strategies in their classrooms. One purpose of this article is to help facilitate educators in convincing their peers that these strategies are worth considering.

## Learning Strategy Use Within Medical Education

While all six of these strategies are well evidenced in the psychology literature, they are not equally used within medical education. Efforts to communicate these learning science principles has been increasing [7, 18]; however, evidence-based practices have also continued to be intermixed with unsupported beliefs, such as the ‘learning styles’ myth [19].

Spaced and interleaved practice have been used in studies spanning many medical specialities [20–25]. These practices can be implemented in a variety of ways, ranging from low-tech—such as flashcards—to more technology assisted—such as scheduling reminder emails to prompt revisiting previously taught topics. The optimal interval for spacing to occur is often discussed as a difficult, yet essential, issue to determine.

Retrieval practice has also been adopted into medical education, particularly with procedural skill training [26–30]. As retrieval practice often requires more effort than re-studying, implementation by the learner strongly relies on their own motivation and adherence to the strategy. Low-stakes quizzes administered by the teacher can help enforce compliance, but then also add to the teacher’s workload and may decrease time available to teach new materials.

Elaboration has also been used well within medical education, particularly alongside self-regulated learning [31–35]. This strategy has been particularly beneficial with topics such as anatomy, where the terms themselves may not be as informative until supplemented by supporting information. Deliberate reflection can also lead to greater elaboration, as it helps foster situational interest and awareness of knowledge gaps that may not be initially apparent.

In some instances, success with learning strategies in medical education has been less than desired. For instance, student engagement statistical power [36] and insufficient pre-existing knowledge [37] have been observed as barriers. These are important concerns and have been observed within the psychology literature over a decade ago, resulting in the desirable difficulties label. As such, a shift in medical education towards transdisciplinary research is critical [38], building transdisciplinary collaborative research teams that include learning science expertise, will provide sufficient consideration for these likely barriers. For instance, many psychology studies have examined factors that influence the optimal spacing interval [39, 40]. While articles such as this are intended to summarise the key features of these learning strategies, these strategies have been studied and refined in hundreds of psychology research studies and associated meta-analyses, which have already examined moderating factors that can influence learning outcomes [41–43].

In conclusion, the implementation of evidence-based learning strategies can significantly enhance the learning outcomes of medical students like Jamie. The six strategies—spaced practice, interleaved practice, retrieval practice, elaboration, dual coding, and using concrete examples—provide a solid foundation for effective learning. However, challenges such as student resistance, conventional educational approaches, and resource constraints can hinder their adoption. By raising awareness of these strategies and addressing the barriers to their implementation, educators can work towards creating an environment that fosters more efficient and lasting learning for their students. As medical education continues to evolve, it is essential to prioritize evidence-based approaches and transdisciplinary collaboration to ensure that the next generation of healthcare professionals is well-equipped to handle the demands of an ever-changing medical landscape.
